# Dexamethasone intravitreal implant as an adjuvant treatment for pediatric patients with Coats’ disease

**DOI:** 10.5935/0004-2749.20230028

**Published:** 2023

**Authors:** Ana Maria Dorado, Almudena De-Pablo-Cabrera, Alicia Muñoz-Gallego, Luis Moreno-García, Ana Barceló-Mendiguchía, Tejada-Palacios Pilar

**Affiliations:** 1 Ophthalmology Department, 12 de Octubre University Hospital, Madrid, Spain

**Keywords:** Retinal telangiectasis, Retinal detachment, Cryotherapy, Dexamethasone, Drug implant, Human, Case report, Telangiectasia retiniana, Descolamento retiniano, Crioterapia, Dexametasona, Implante de medicamento, Humano, Relato de caso

## Abstract

We report two cases of stage 3A unilateral Coats’ disease in pediatric patients. In both cases, disease control was achieved using a dexamethasone intravitreal implant in addition to other treatments. The treatment improved visual acuity in one patient and prevented the worsening of the decline in visual acuity in the other patient during follow-up periods of 7 and 3 years, respectively. One of the patients presented an increase in intraocular pressure, which was controlled with topical antiglaucoma medication, but developed a cataract that required surgery. In conclusion, dexamethasone intravitreal implant may be a useful adjuvant treatment to consider in some pediatric cases with Coats’ disease.

## Abstract (pt)

Relatamos dois casos de doença de Coats em estágio 3A unilateral em pacientes pediátricos. Em ambos os casos, o controle da doença foi obtido com implante intravítreo de dexametasona, além de outros tratamentos, com melhora da acuidade visual em um caso e sem piora da visão no outro, durante um período de acompanhamento de 7 e 3 anos. Um dos casos apresentou elevação da pressão intraocular controlada com medicação antiglaucoma tópica e desenvolveu catarata que exigiu cirurgia. Em conclusão, o implante intravítreo de dexametasona pode ser um tratamento adjuvante útil a ser considerado em alguns casos pediátricos com doença de Coats.

## INTRODUCTION

Coats’ disease is an idiopathic exudative retinopathy characterized by retinal telangiectasis, aneurysms, and capillary non-perfusion, and associated with intraretinal and subretinal exudation, which frequently progresses to exudative retinal detachment (RD). Its most common classification was proposed by Shields et al. and is based on funduscopic findings^(1)^, which usually lead to diagnosis^(1,2)^. Among the pathologies that can simulate Coats’ disease are retinoblastoma, retinal vasoproliferative tumor, familial exudative retinopathy, retinal capillary hemangioblastoma, and familial retinal arterial macroaneurysm^(1,2)^.

Depending on the severity of Coats’ disease, its conventional treatment includes photocoagulation, cryotherapy, and surgery^(1,2)^. Other treatment modalities such as intravitreal injection of anti-vascular endothelial growth factor (VEGF)^(3)^ or corticosteroids^(2)^ may also be applied as adjuvant treatments. The efficacy of dexamethasone intravitreal implant (Ozurdex, Allergan Pharmaceuticals, Irvine, CA, USA) has also been reported recently^(4,5,6,7)^.

We inform about our experience with the use of dexamethasone intravitreal implant as an adjuvant treatment to photocoagulation and cryotherapy in two pediatric cases of stage 3A Coats’ disease.

## CASE REPORTS

### Case 1

**Figure 1:**
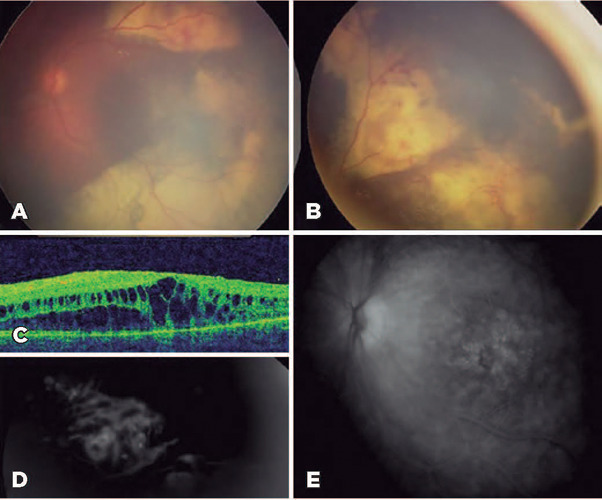
Images of case 1 at the time of diagnosis. The retinography image shows a subtotal exudative retinal detachment with abundant intraretinal lipid exudates, a subretinal nodule at the superior temporal arcade (A), and peripheric telangiectasias and vascular anomalies (B). The optical coherence tomography (OCT) image shows a macular edema (C). The fuorescein angiogram shows late leakage from the telangiectatic vessels (D) and macular edema (E).

**Figure 2:**
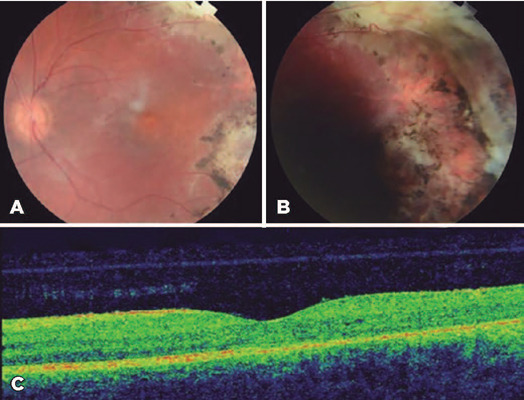
Images of case 1 after treatment with three dexamethasone intravitreal implants as adjuvant treatment, cryotherapy, and laser photocoagulation. The retinography image shows the treated retina, scarring from cryotherapy and laser photocoagulation, resolution of telangiectasia and lipid exudates (A, B). The optical coherence tomography image shows resolution of the macular edema (C).

An 8-year-old boy was referred to us because of visual loss in his left eye (OS). The best-corrected visual acuity (BCVA) was 20/20 in his right eye (OD) and 20/200 in his OS. In his OS, fundoscopy revealed an inferotemporal exudative RD, retinal telangiectasis, abundant subretinal exudation, and a fibrous nodule. In addition, optical coherence tomography (OCT) confirmed the exudative RD with severe macular edema (ME), and fluorescein angiography revealed findings consistent with Coats’ disease (Figure 1). He was diagnosed as having stage 3A Coats’ disease in OS and treated with an intravitreal injection of ranibizumab and cryotherapy, without improvement. Thus, we de decided to treat him with a dexamethasone intravitreal implant. Two weeks later, the exudative RD resolved. A month later, his intraocular pressure (IOP) increased to 24 mmHg but was well controlled with topical timolol therapy. Six months later, he had a recurrence, and a dexamethasone intravitreal implant was injected followed by laser photocoagulation, thereby achieving complete resolution. Six months later, he developed a cataract that required surgery. Four months later, another recurrence occurred; thus, a third dexamethasone intravitreal implant was administered followed by laser photocoagulation, which achieved complete resolution. Seven years later, he continues to be in regression, and his BCVA was 20/50 in OS (Figure 2).

### Case 2

**Figure 3:**
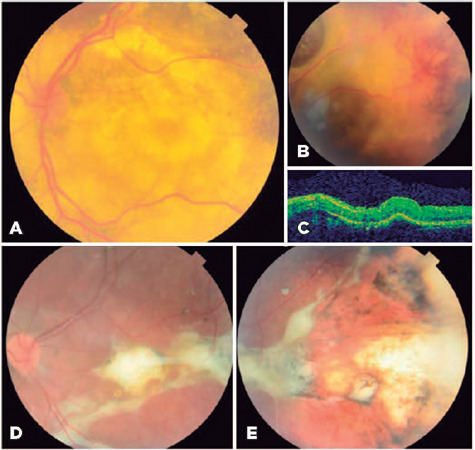
Images of case 2. The retinography image shows a subtotal exudative retinal detachment with abundant lipid exudates (A), and retinal telangiectasis and vascular anomalies in periphery (B). The optical coherence tomography image shows retinal detachment and a subfoveal fbrous nodule (C) at the time of diagnosis. The retinography image shows complete resolution of the retinal detachment with a persistent subfoveal fbrous nodule (D) and resolution of telangiectasias and lipid exudates (D, E) after treatment with a dexamethasone intravitreal implant as adjuvant treatment and two sessions of cryotherapy.

A 4-year-old girl was referred to us because of visual loss in her OS. The BCVA was 20/20 in OD and hand movement in OS. In her OS, fundoscopy revealed an extensive exudative RD with retinal telangiectasis and abundant lipidic exudation, and OCT confirmed an inferotemporal exudative RD with a subfoveal fibrous nodule (Figure 3). She was diagnosed as having stage 3A Coats’ disease in OS and treated with a dexamethasone intravitreal implant and cryotherapy; thereby, complete resolution of the RD was achieved, but the retinal telangiectasis did not completely regress. A second cryotherapy session was performed, which achieved complete regression (Figure 3). Three years later, she was stable, with a BCVA of hand movement.

Table 1 summarizes the cases.

**Table 1:** Summarizes the cases

Case	Sex	Age (years)	Eye affected	Disease stage	Initial BCVA (Snellen)	Final BCVA	Fundus	Number of DII	Treatment	Complications of DII	Follow-up time (years)	Results
1	Male	8	Left	3A	20/200	20/50	Retinal telangiectasis, subretinal exudation, fibrous nodule, partial exudative retinal detachment	3	Intravitreal ranibizumab + Cryotherapy DII DII + Laser photocoagulation Facoemulsification DII + Laser photocoagulation	Elevated IOP Cataract	7	No significant improvement Resolution of ERD Regression of telangiectasis + resolution of ERD Regression of telangiectasis + resolution of ERD
2	Female	4	Left	3A	HM	HM	Retinal telangiectasis, subretinal exudation, subfoveal, fibrous nodule, partial exudative retinal detachment	1	DII + Cryotherapy (2 sesions).		3	Resolution of ERD + regression of telangiectasis

BCVA= best corrected visual acuity; HM= hand movements; DII= dexamethasone intravitreal implant; IOP= intraocular pressure; ERD= exudative retinal detachment.

## DISCUSSION

Several authors have reported high levels of VEGF in eyes with Coats’ disease^(8)^. Corticosteroids reduce intraocular inflammation and stabilize the hematoretinal barrier, decreasing vascular permeability, partly owing to the inhibition of VEGF. For this reason, they constitute part of the therapeutic arsenal for retinal vascular diseases^(9)^.

Adult Coats’ disease has been reported in patients with exudative RD treated with a dexamethasone intravitreal implant and posterior photocoagulation, which achieved resolution of the RD with BCVA improvement and without recurrence^(7)^.

Dexamethasone intravitreal implants are currently used as non-approved treatments in some pediatric ocular pathologies^(10)^. Use of a dexamethasone intravitreal implant has been reported in children with Coats’ disease. Lei and Lam^(4)^ reviewed four pediatric cases treated with dexamethasone intravitreal implants for ME of different causes. One of the cases was a 4-year-old boy with Coats’ disease who had been previously treated with laser photocoagulation and multiple anti-VEGF injections without success. After three injections of a dexamethasone intravitreal implant, his ME diminished and BCVA improved. He developed a cataract, and his increased IOP was controlled with a topical hypotensive medication. Saatci et al.^(5,6)^ reported three cases of children with stage 3A and 2B Coats’ disease treated with dexamethasone intravitreal implant and laser photocoagulation. In all the cases, the macular exudates and subretinal fluid were resolved, and the BCVA eventually improved. One of the cases had previously received 5 monthly intravitreal injections of anti-VEGF and laser photocoagulation, which were not effective. Two patients developed an increase in IOP 5 weeks after the dexamethasone intravitreal implant, which was controlled with topical hypotensive medication. One patient developed a cataract that required surgery.

In both cases presented in this report, treatment with dexamethasone intravitreal implant completely resolved the subretinal fluid and improved the exudates, allowing photocoagulation or cryotherapy for the retinal telangiectasis, which had a good final result. One of the patients developed an increase in IOP, which was well controlled with topical hypotensive medication. After the second implant, a cataract developed, which required surgery, and a final BCVA of 20/50 was achieved. The second patient did not present any side effects from a single dexamethasone intravitreal implant. However, the BCVA did not improve because of a fibrotic subfoveal nodule, which was already present at the time of diagnosis.

Coats’ disease can sometimes be highly aggressive and requires the use of multiple therapies to control the inflammation. Cases with a poor evolution may even require enucleation^(1)^. Considering this, we believe that in selected cases with massive exudation, use of a dexamethasone intravitreal implant is an effective therapeutic option as adjuvant treatment to facilitate laser photocoagulation or cryotherapy. However, it must be kept in mind that it is a non-approved drug for pediatric patients owing to its potential side effects such as ocular hypertension and cataracts, which can be especially problematic in children.

In our experience, the main benefit of a dexamethasone intravitreal implant is that it allows effective retinal photocoagulation or cryotherapy by eliminating subretinal fluid and prevention of macular structural irreversible damage as a consequence of maintained ME.

More studies are needed to establish the efficacy and safety of dexamethasone intravitreal implants in advanced stages of pediatric Coats’ disease.
